# Associations between alcohol intake and diabetic retinopathy risk: a systematic review and meta-analysis

**DOI:** 10.1186/s12902-020-00588-3

**Published:** 2020-07-17

**Authors:** Chen Chen, Zhaojun Sun, Weigang Xu, Jun Tan, Dan Li, Yiting Wu, Ting Zheng, Derong Peng

**Affiliations:** 1 Shanghai Jing ‘an District Jiangning Road Community Health Service Center, Shanghai, China; 2 Shanghai Jing ‘an District Pengpuxincun Community Health Service Center, No.15 Pingshun Road, Jing ‘an District, Shanghai, 200435 China

**Keywords:** Alcohol, Diabetic retinopathy, Meta-analysis

## Abstract

**Background:**

Some previous studies have reported inconsistent results on the association between alcohol intake and diabetic retinopathy (DR) risk. This study aimed to evaluate the potential effects of alcohol intake on subsequent DR risk using a meta-analytic approach.

**Methods:**

Three electronic databases (PubMed, EmBase, and the Cochrane library) were systematically searched for observational studies from their inception till November 2019. The pooled odds ratio (OR) with 95% confidence interval (CI) were applied for the summary effect estimate using a random-effects model.

**Results:**

A total of 15 studies (5 cohort studies, 4 case-control studies, and 6 cross-sectional studies) with 37,290 participants and 12,711 DR cases were selected for the final meta-analysis. The pooled OR indicated no significant association between alcohol intake and DR risk (OR: 0.91; 95%CI: 0.78–1.06; *P* = 0.225), irrespective of the studies being pooled cohort (OR: 0.95; 95%CI: 0.66–1.36; *P* = 0.761), case-control (OR: 0.97; 95%CI: 0.77–1.23; *P* = 0.818), or cross-sectional (OR: 0.86; 95%CI: 0.69–1.08; *P* = 0.190) ones. However, this association might have been affected by the type of diabetes mellitus and the adjusted status.

**Conclusion:**

The results of this study showed that the potential impact of alcohol intake on DR risk may differ according to the type of diabetes mellitus and adjusted status. Further large-scale, prospective cohort studies should be conducted to verify the findings of this study and to evaluate DR risk in relation to the dose and type of alcohol intake.

## Background

Globally, diabetes mellitus (DM) has been rapidly increasing and is estimated to have affected about 422 million people and caused 1.6 million deaths in 2014 [1]. Diabetic patients experience progressive changes in their metabolic and inflammatory indices and several inflammatory markers [2–4]. Microvascular abnormalities and eye-related complications are most common in DM patients [5, 6]. Diabetic retinopathy (DR) is one of the most severe complications of DM and accounts for nearly 40% of DM complications in patients aged ≥40 years. Patients with DR have an increased risk of permanent visual impairment, and their quality of life is adversely affected [7–9]. A study reported that the prevalence of DR exceeds 75% in patients with DM for more than 20 years [10]. DR is the leading cause of impaired vision and blindness in DM patients and accounts for 4.8% of blindness cases worldwide [11, 12]. Therefore, identifying potential risk factors for the progression of DR is important in DM patients.

Several studies have identified some of the potential risk factors for the progression of DR. A meta-analysis conducted by Song et al. contained 31 studies and found that insulin treatment, elevated fasting blood glucose levels, and high glycosylated hemoglobin concentrations are associated with an increased risk of DR in Chinese diabetic patients [13]. Moreover, several other risk factors, including hyperhomocysteinemia [14], vitamin D deficiency [15], obstructive sleep apnea [16], and obesity [17] have been demonstrated to be associated with an increased risk of DR. The investigating the potential role of alcohol intake on the risk of DR with an important public health implications owing to alcohol was the most widely consumed beverages. Therefore, to clarify the role of alcohol intake plays in DR is particularly important, as it not defined in general and DM populations. In this study, we attempted a large-scale examination of the available observational studies to determine the association between alcohol intake and DR risk. Stratified analyses were also conducted according to the study design.

## Methods

### Data sources, search strategy, and selection criteria

This review was conducted and reported according to the Preferred Reporting Items for Systematic Reviews and Meta-Analysis Statement issued in 2009 [18]. Observational studies that investigated the association of alcohol intake with DR were included in this study, without restrictions on language and published status. Three electronic databases PubMed, EmBase, and the Cochrane library were systematically searched throughout November 2019 using “alcohol” and “diabetic retinopathy” as the core search terms. Manual searches were also performed for the reference lists of the retrieved studies to identify any new eligible study.

A standardized approach was applied by two of the authors for the literature search and study selection, with any disagreement between them resolved by a group discussion until a consensus was reached. The inclusion criteria of this study were as follows: (1) Study design: observational studies, including cohort, case-control, and cross-sectional studies; (2) Participants: there were no restrictions, with the inclusion of general population as well type 1 DM, type 2 DM, or mixed patients; (3) Exposure: alcohol intake; and (4) Outcomes: studies reporting an effect estimate and 95% confidence intervals (CIs) for comparisons of high and low alcohol intake on the risk of DR. The maximally adjusted results were selected if the study reported several adjusted effect estimates.

### Data collection and quality assessment

Data collection and quality assessment were performed by two authors, and any inconsistency was settled by an additional author by referring to the original article. The following data items were collected: first author’s surname, publication year, study design, country, sample size, male participant percentage, mean age, number of cases, DR diagnosis, DR definition, population status, exposure definition, effect estimate and its 95% CI, and covariates in the fully adjusted model. The quality of identified studies was assessed using Newcastle–Ottawa Scale (NOS), which has already been partially validated for assessing the quality of observational studies in meta-analyses [19]. NOS comprises a star system that includes selection (four items), comparability (one item), and outcome (three items) categories; the number of stars awarded ranges from 0 to 9.

### Statistical analysis

The association between alcohol intake and DR risk on the basis of effect estimate and corresponding 95%CIs in each study as well as the pooled odds ratio (OR) with 95%CI was calculated using the random-effects model [20, 21]. *I*^*2*^ index and Q statistic was applied to assess heterogeneity among the studies, and *I*^*2*^ > 50.0% or *P* < 0.10 was considered as significant heterogeneity [22, 23]. The robustness of pooled conclusion was evaluated using a sensitivity analysis [24]. Subgroup analyses were also conducted based on countries, publication year, population status, adjusted status, and study quality according to the study design. The *P* value between the subgroups was assessed using an interaction test [25]. Publication bias was assessed using the funnel plot and Egger’s and Begg’s tests [26, 27]. All reported *P* values are two-sided, and P values < 0.05 were considered significant for all the included studies. Statistical analyses were performed using STATA software (version 12.0; Stata Corporation, College Station, TX, USA).

## Results

### Literature search

**Fig. 1:**
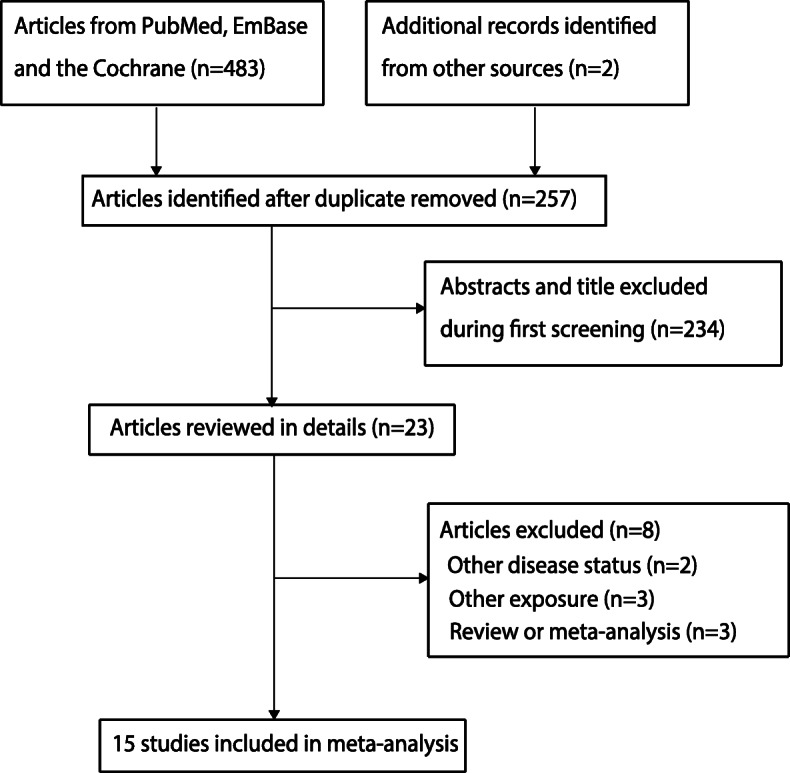
Flow diagram of the literature search and study selection process

A total of 483 articles were identified in our initial electronic searches; 460 studies were excluded due to duplication and irrelevancy. A total of 23 potentially eligible studies were selected for further full-text evaluations, and 8 studies were excluded due to other disease status (*n* = 2), other exposure (*n* = 3), and the study being a review or meta-analysis (n = 3). Eventually, 15 observational studies were selected for the final quantitative analysis [28–42]. A manual search for the reference lists yielded two studies, and these two studies were included in the initial electronic searches. Figure 1 presents the study selection process; the baseline characteristics of the included studies and participants are summarized in Table 1.

**Table 1:** Baseline characteristics of the selected studies

Study	Publication year	Study design	Country	Sample size	Percent of male (%)	Mean age (years)	Number of cases	DR diagnosis	DR definition	Diabetes	Exposure Definition	Adjustment/matched	NOS score
Young [28]	1984	Cohort	UK	296	100.0	20.0–59.0	66	Fundoscopic	Four Grades	Mixed	≤10 measures/ week, > 10 measures/week	Crude	6
Moss [29]	1994	Cohort	USA	916	NA	≥ 21.0	238	Fundus photographs	ETDRS	Mixed	Average loz/ day increase	Age, sex, HbA1c, retinopathy	8
Kohner [30]	1998	Case control	UK	2964	58.4	25.0–65.0	1102	Retinal photography	ETDRS	T2DM	None, occasional, regular, heavy	Crude	6
Rasmidatta [31]	1998	Case control	Thailand	198	NA	60.5	63	Fundoscopic examinations	Three grades	T2DM	Nondrinker, drinker, not regular drinker	HbA1c, cholesterol, triglyceride, HDL, BP	6
Giuffrè [32]	2004	Case control	Italy	132	38.6	≥ 40.0	45	Fundus examination	ETDRS	Mixed	None, 1–19 years, 20 years or more	Crude	6
Hirai [33]	2007	Cross-sectional	USA	537	50.1	45.3	309	Retinal photography	ETDRS	T1DM	Alcohol/No alcohol	Crude	4
Beulens [34]	2008	Cross-sectional	Europe	3250	29.7	15.0–60.0	304	Retinal photographs	Three grades	T1DM	0 g/week, 0.0–4.9 g/week, 5.0–29.9 g/ week, 30.0–69.9 g/week, 70.0–209.9 g/week, ≥210 g/week	Age, sex, centre, duration of illness, systolic BP, physical activity, smoking, BMI, presence of cardiovascular disease and HbA1c	7
Xu [35]	2009	Cohort	China	4141	43.4	≥ 40.0	366	Fundus photographs	NA	General	Consumers, non-consumers	BMI, HDL, LDL, arterial hypertension	7
Lee [36]	2010	Cohort	14 countries	1239	60.7	55.0–81.0	640	Retinal photography	ETDRS	T2DM	0, drinks /week 1–14, drinks/week > 14 drinks /week	Age, sex, HbA1c, systolic BP, duration of diabetes, BMI, cigarette smoking, ethnicity	8
Yang [37]	2013	Cross-sectional	Korea	978	54.1	≥ 19.0	112	Fundus examination	ETDRS	Mixed	≥4 alcoholic drinks/week, < 3 drinks/week	Age, gender, smoking status, regular exercise, BMI, serum total cholesterol, serum triglyceride, serum HDL cholesterol, anti-lipid drug use	6
Jongsareejit [38]	2013	Cross-sectional	Thailand	933	NA	59.5	214	Indirect ophthalmoscope	International scales	T2DM	No, ever, current	Gender, age, diastolic BP, waist, total cholesterol, HDL, ccular perfusion pressure	5
Harjutsalo [39]	2014	Cross-sectional	Finland	3608	52.6	28.9–46.8	1191	Retinal photography	NA	T1DM	Heavy drinker light drinker	Crude	3
Fenwick [40]	2015	Cross-sectional	Australia	395	64.1	≥ 18.0	235	Fundus photography	ETDRS	T2DM	None, moderate, high	Education, income, language spoken at home, country of birth, lipid lowering drugs, hypertension drugs	6
Tseng [41]	2015	Cohort	China	573	61.8	58.9	91	Funduscopic	Three grades	T2DM	Drinker, no-drinker	Crude	6
Martín-Merino [42]	2016	Case control	UK	17,130	55.9	All stages	7735	Computerized records	NA	T2DM	0–1 units/week 2–21 units/ week 22–34 units/week ≥35 units/week	Sex, age at index date, diabetes duration, primary care practitioner visits, referrals and hospitalizations, smoking, first HbA1c; systolic BP, glaucoma; cataracts, or lens extraction, HDL and triglycerides, and hypoglycaemic agents, including oral hypoglycaemic drugs and insulin	7

### Study characteristics

Of the 15 included studies, 5 were cohort, 4 were case-control, and the remaining 6 were cross-sectional studies. The studies were published between 1984 and 2016, and the participants in the individual studies ranges from 132 to 17,130. A total of 11 studies were conducted in Western countries, and the remaining 4 studies were conducted in Eastern countries. Three studies included type 1 DM patients, seven included type 2 DM patients, four included both type 1 and type 2 DM patients, and the remaining study included the general population. Nine studies reported that effect estimates were adjusted for potential covariates, and the remaining six studies reported crude effect estimates. Studies were assessed using NOS: two studies were awarded 8 stars, three studies were awarded 7 stars, seven studies were awarded 6 stars, one study was awarded 5 stars, 1 study was awarded 4 stars, and the remaining study was awarded 3 stars.

### Meta-analysis

**Fig. 2:**
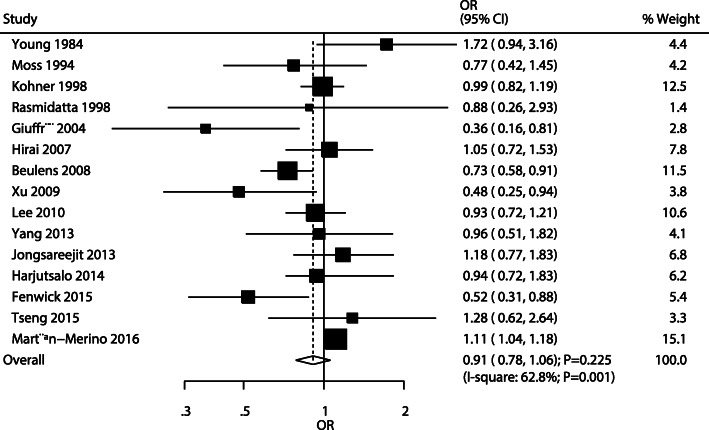
The pooled odds ratio for the association of alcohol intake with diabetic retinopathy risk

**Fig. 3:**
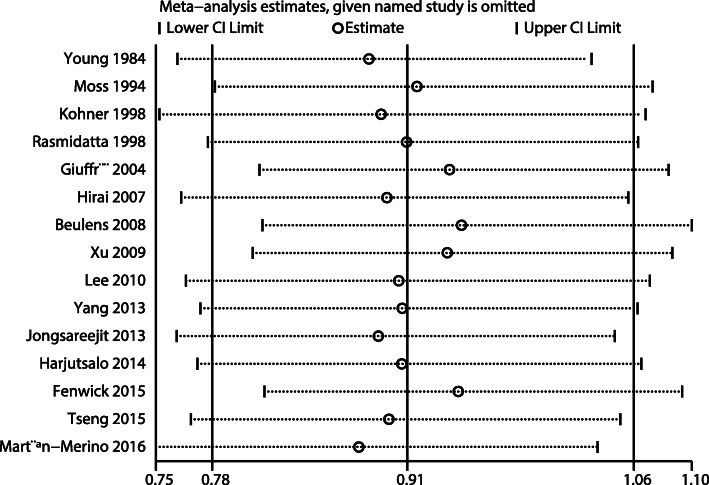
Sensitivity analysis

After pooling all the included studies, the pooled OR indicated no significant association between alcohol intake and DR risk (OR: 0.91; 95%CI: 0.78–1.06; *P* = 0.225; Fig. 2), and significant heterogeneity was observed across the studies (*I*^*2*^ = 62.8%; *P* = 0.001). The conclusion was not altered by sequentially excluding individual studies (Fig. 3). When stratified by study design, no significant associations were observed irrespective of the studies being pooled cohort (OR: 0.95; 95%CI: 0.66–1.36; *P* = 0.761), case-control (OR: 0.97; 95%CI: 0.77–1.23; *P* = 0.818), or cross-sectional (OR: 0.86; 95%CI: 0.69–1.08; *P* = 0.190) ones. Sensitivity analyses were also conducted according to the study design, showing that alcohol intake was not associated with DR risk in cohort and cross-sectional studies, whereas a potential significant association was observed in case-control studies (Additional file 1, Additional file 2 and Additional file 3).

### Subgroup analysis

Subgroup analyses were conducted to evaluate the association between alcohol intake and DR risk according to the study design (Table 2). When stratified analyses were conducted for cohort studies, alcohol intake was found to be associated with a reduced DR risk if the study included general population; furthermore, the association between alcohol intake and DR risk could be affected by the adjusted status and study quality. When stratified analyses were conducted for case-control studies, alcohol intake was found to be associated with an increased DR risk if the analysis included pooled studies published in or after 2010, studies on type 2 DM patients, studies reporting adjusted effect estimates, and studies with high quality; however, alcohol intake was associated with a reduced DR risk if studies included both type 1 and type 2 DM patients. Moreover, the association of alcohol intake with DR risk could be affected by the population status. When stratified analyses were conducted for cross-sectional studies, alcohol intake was found to be associated with a reduced DR risk if the pooled studies were of high quality.

**Table 2:** Subgroup analyses according to study design

Study design	Factors	Group	OR and 95%CI	P value	Heterogeneity (%)	P value for Q test	P value between subgroups
Cohort studies	Countries	Western	1.04 (0.70–1.53)	0.855	50.5	0132	0.318
		Eastern	0.77 (0.30–2.03)	0.603	73.9	0.050	
	Publication year	Before 2010	0.87 (0.42–1.80)	0.703	75.3	0.018	0.722
		2010 or after	0.96 (0.76–1.23)	0.771	0.0	0.416	
	Population	T2DM	0.96 (0.76–1.23)	0.771	0.0	0.416	0.086
		Mixed	1.15 (0.52–2.54)	0.722	69.7	0.069	
		General	0.48 (0.25–0.93)	0.030	–	–	
	Adjusted status	Yes	0.77 (0.53–1.11)	0.162	41.3	0.182	0.024
		No	1.52 (0.96–2.42)	0.076	0.0	0.540	
	Study quality	High	0.77 (0.53–1.11)	0.162	41.3	0.182	0.024
		Low	1.52 (0.96–2.42)	0.076	0.0	0.540	
Case control studies	Countries	Western	0.97 (0.75–1.24)	0.792	76.5	0.014	0.729
		Eastern	0.88 (0.26–2.95)	0.836	–	–	
	Publication year	Before 2010	0.71 (0.36–1.41)	0.332	64.9	0.058	0.086
		2010 or after	1.11 (1.04–1.18)	0.001	–	–	
	Population	T2DM	1.10 (1.03–1.16)	0.003	0.0	0.490	0.007
		Mixed	0.36 (0.16–0.81)	0.014	–	–	
	Adjusted status	Yes	1.11 (1.04–1.18)	0.001	0.0	0.707	0.093
		No	0.65 (0.24–1.72)	0.383	82.4	0.017	
	Study quality	High	1.11 (1.04–1.18)	0.001	–	–	0.086
		Low	0.71 (0.36–1.41)	0.332	64.9	0.058	
Cross-sectional studies	Countries	Western	0.79 (0.61–1.03)	0.080	46.9	0.130	0.088
		Eastern	1.11 (0.77–1.58)	0.583	0.0	0.599	
	Publication year	Before 2010	0.85 (0.60–1.20)	0.354	62.0	0.105	0.532
		2010 or after	0.87 (0.61–1.24)	0.451	48.4	0.121	
	Population	T1DM	0.85 (0.67–1.08)	0.187	33.5	0.223	0.896
		T2DM	0.79 (0.36–1.77)	0.573	82.2	0.018	
		Mixed	0.96 (0.51–1.81)	0.900	–	–	
	Adjusted status	Yes	0.80 (0.59–1.10)	0.170	54.4	0.087	0.144
		No	1.01 (0.75–1.35)	0.973	00	0.718	
	Study quality	High	0.73 (0.58–0.91)	0.006	–	–	0.114
		Low	0.92 (0.70–1.20)	0.538	36.9	0.175	

### Publication bias

**Fig. 4:**
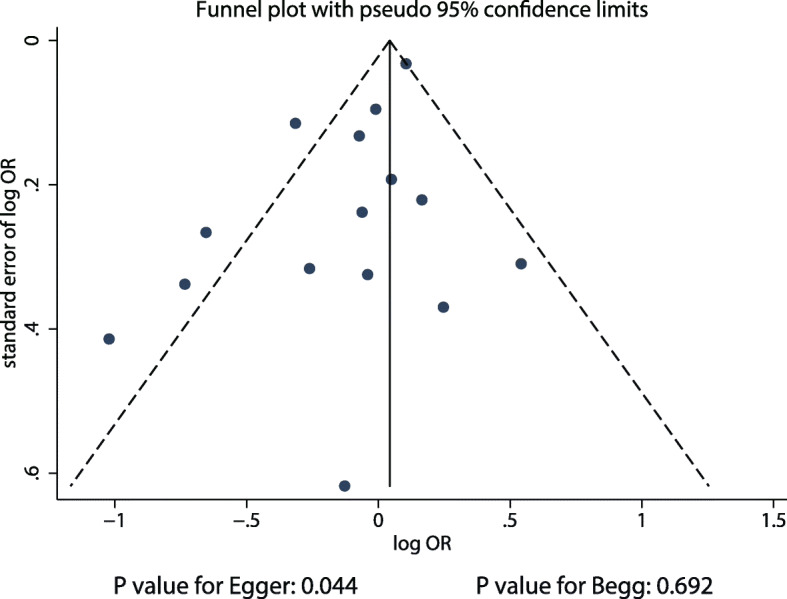
Publication bias

The publication bias could not be ruled out by reviewing the funnel plot for the association between alcohol intake and DR risk (Fig. 4). Although the Begg’s test indicated no significant publication bias (*P* = 0.692), the Egger’s test suggested significant publication bias (*P* = 0.044). The conclusions were unaltered after adjustments for publication bias through the trim and fill method [43].

## Discussion

This study was conducted on the basis of previously published observational studies, and it evaluated the association of alcohol intake with DR risk. This quantitative meta-analysis included 37,290 participants and 12,711 DR cases from 5 cohort studies, 4 case-control studies, and 6 cross-sectional studies across a wide range of participant characteristics. The findings of this study show no significant association between alcohol intake and DR risk, irrespective of the studies being pooled cohort, case-control, or cross-sectional ones. Sensitivity analysis suggested potential beneficial effects of alcohol intake on DR risk in case-control studies. Finally, the association of alcohol intake with DR risk according to study design varied when the studies were stratified by countries, publication year, population status, adjusted status, and quality.

A meta-analysis conducted by Zhu et al. included a total of 15 studies and found that alcohol intake was not associated with DR risk. Interestingly, wine or sherry intake was associated with a reduced DR risk [44]. They attributed the results to the potential protective effects of low to moderate alcohol intake on the risk of DM and cardiovascular disease [45, 46]. However, the inflammatory response and oxidative stress could be affected by alcohol and are significantly associated with DR risk [47, 48]. The stratified analyses from the previous meta-analysis were mixed owing to, studies with various designs, and the results of such stratified analyses are unreliable. Therefore, the present study may correct the inappropriate results reported by such stratified analyses.

Although no significant association between alcohol intake and DR risk was observed in most of the studies included in our meta-analysis, many of these studies reported inconsistent results. The Casteldaccia Eye Study found that the duration of alcohol intake between 1 and 19 years was not associated with DR risk, whereas alcohol intake for ≥20 years was associated with a reduced DR risk [32]. Beulens et al. reported that moderate alcohol intake was associated with a reduced risk of microvascular complications among type 1 DM patients [34]. The Beijing Eye Study suggested that alcohol intake was associated with a reduced DR risk in general population [35]. A study conducted by Fenwick et al. found that moderate white and fortified wine intake was correlated with DR risk among type 2 DM patients [40]. They pointed out the beneficial effects induced by alcohol intake due to increase in high-density lipoprotein levels, reduction in platelet aggregation, and decrease in fibrinogen levels [49]. However, a case-control study in a UK primary care setting indicated that alcohol intake was associated with an increased DR risk among type 2 DM patients [42]. A possible reason for this could be the moderate to heavy rate at which alcohol was consumed by the participants of that study, which has been associated with an increased DR risk.

The results of the subgroup analyses showed that the association of alcohol intake with DR risk is multifaceted when stratified by countries, publication year, population status, adjusted status, and study quality. First, we found that the association of alcohol intake with DR risk persisted even after stratification by countries, irrespective of the studies being cohort, case-control, or cross-sectional ones. However, the heterogeneity remained and was not fully explained. Second, we found that alcohol intake was associated with an increased DR risk in studies published in or after 2010 when stratified by case-control cohorts; this result was obtained from only one study and has been previously identified [42]. Third, we found that alcohol intake was associated with an increased DR risk if only type 2 DM patients were included, whereas the risk was significantly reduced if both type 1 and type 2 DM patients were included in stratified case-control cohorts. This could be due to the study conducted by Martín-Merino et al. contributing a large weight to the overall analysis [42]. A similar result was observed when the studies were stratified by adjusted status. Finally, when the studies were pooled by design as case-control or cross-sectional studies with high quality, conflicting results were observed. However, this observation was obtained from only one study, and the conclusions were not reliable.

There are several limitations to this study. First, most of the included studies (10/15) were designed as case-control or cross-sectional studies, making it difficult to distinguish cause-and-effect relationships. Second, the drinking habits and other lifestyle factors after the diagnosis of DM may have changed, altering the effects of alcohol intake on DR risk and biasing the results. Third, the adjusted status and included covariates were different across the included studies, which could affect the reliability of the pooled conclusion. Forth, stratified analyses according to sex, the dose and type of alcohol intake were not conducted owing to mostly included studies did not report these data. Finally, the inherent limitations of traditional meta-analysis, including publication bias and study level-based analysis, affect the reliability of conclusion and restrict the results of detailed analyses.

## Conclusions

In conclusion, the findings of this study suggest no significant association between alcohol intake and DR risk. Moreover, this lack of association might have been affected by population status, adjusted status, and study quality. This association should be verified in further large-scale, prospective studies, and DR risk in relation to the dose and type of alcohol intake should also be explored.

## Supplementary information

**Additional file 1.** Sensitivity for cohort studies.

**Additional file 2.** Sensitivity for case control studies.

**Additional file 3.** Sensitivity for cross-section studies.

## Data Availability

The datasets used and/or analysed during the current study are available from the corresponding author on reasonable request.

## Abbreviations

DR: Diabetic retinopathy
OR: Odds ratio
CI: Confidence interval
DM: Diabetes mellitus
NOS: Newcastle–Ottawa Scale
