# Supplementary figures and images for: Associations between alcohol intake and diabetic retinopathy risk: a systematic review and meta-analysis
Source: BMC Endocr Disord. 2020 Jul 17;20:106. doi: 10.1186/s12902-020-00588-3 (PMC7368775; doi:10.1186/s12902-020-00588-3)

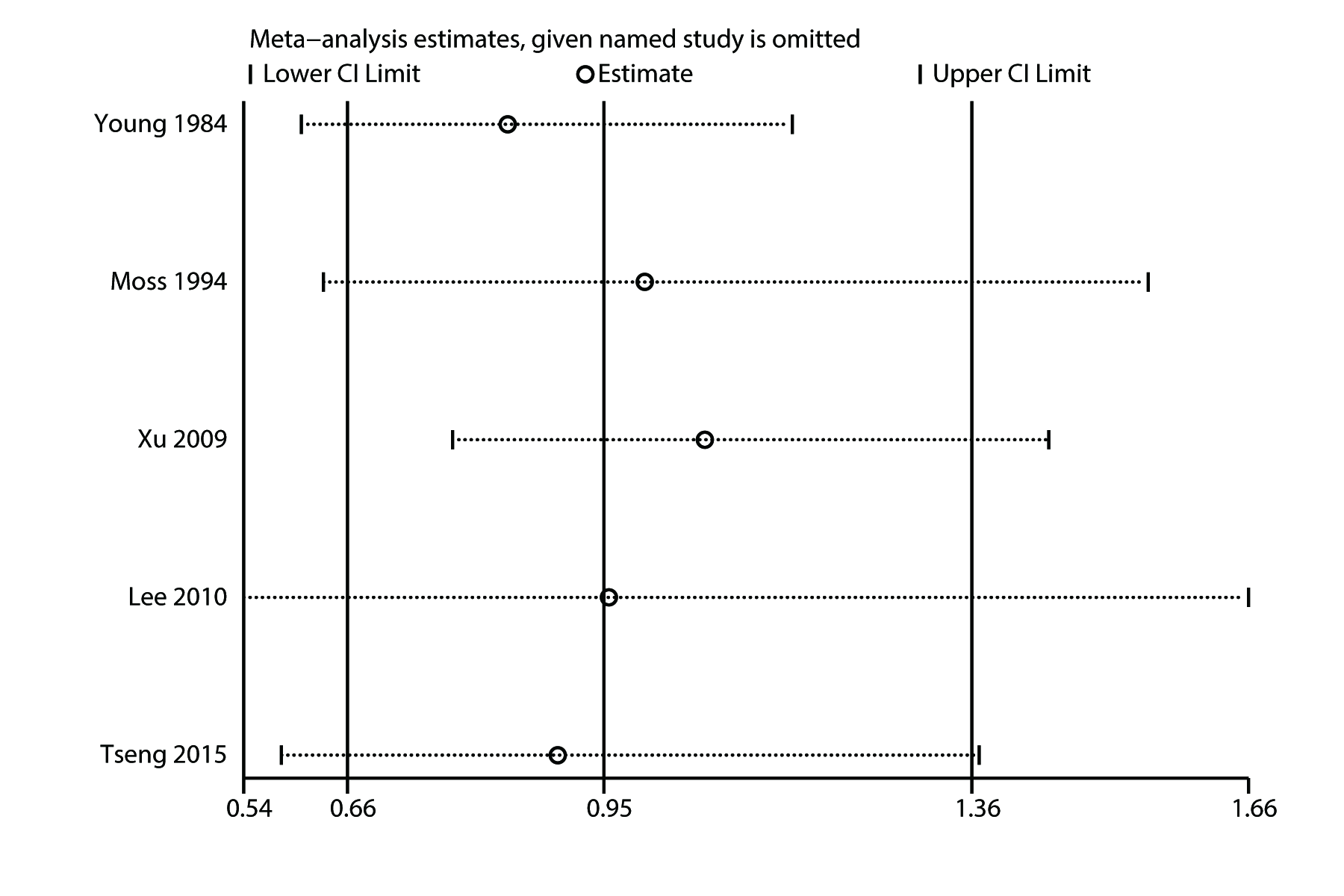

Supplement: Supplementary file 1 — Additional file 1. Sensitivity for cohort studies. [file 12902_2020_588_MOESM1_ESM.tif]

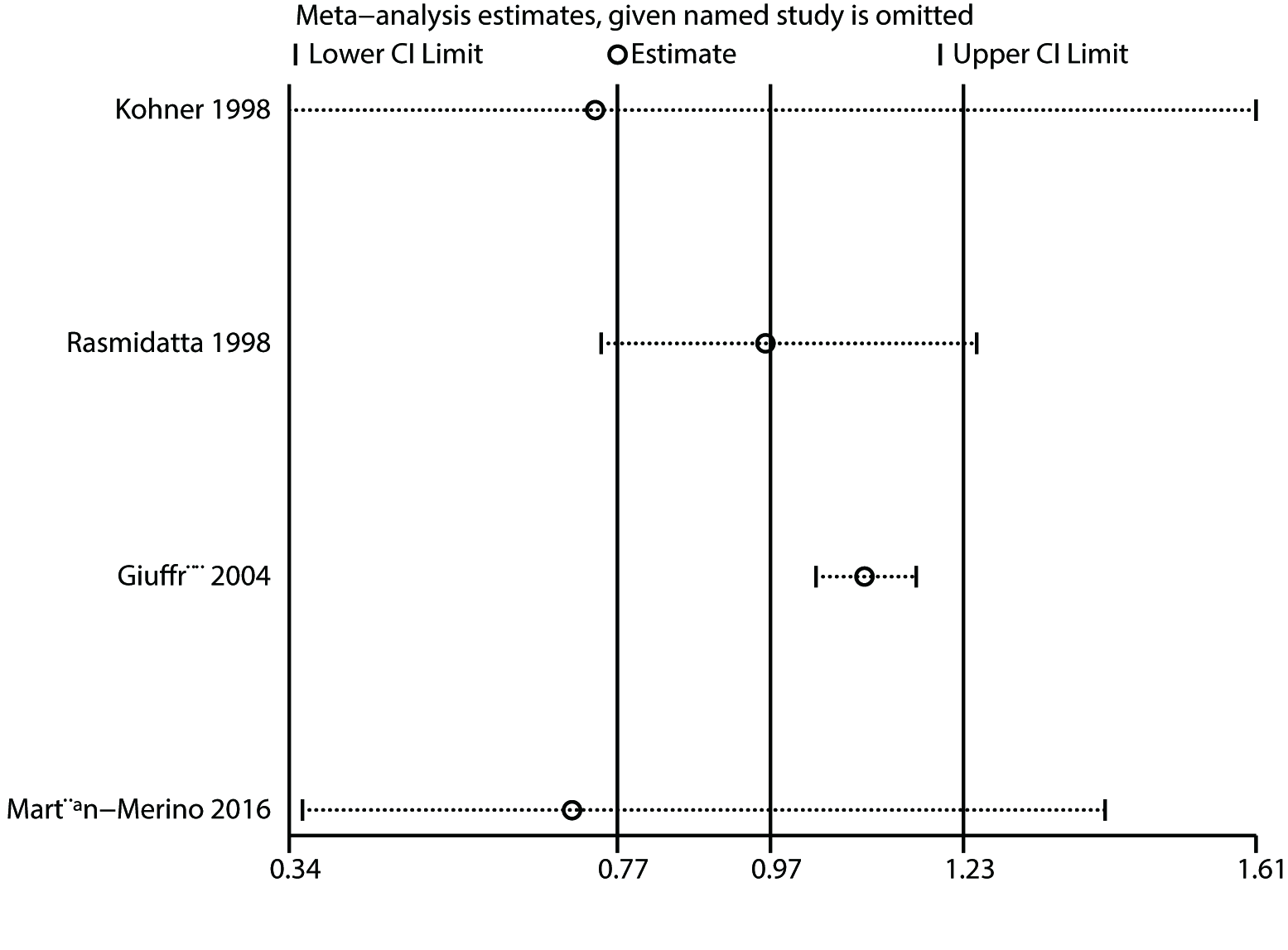

Supplement: Supplementary file 2 — Additional file 2. Sensitivity for case control studies. [file 12902_2020_588_MOESM2_ESM.tif]

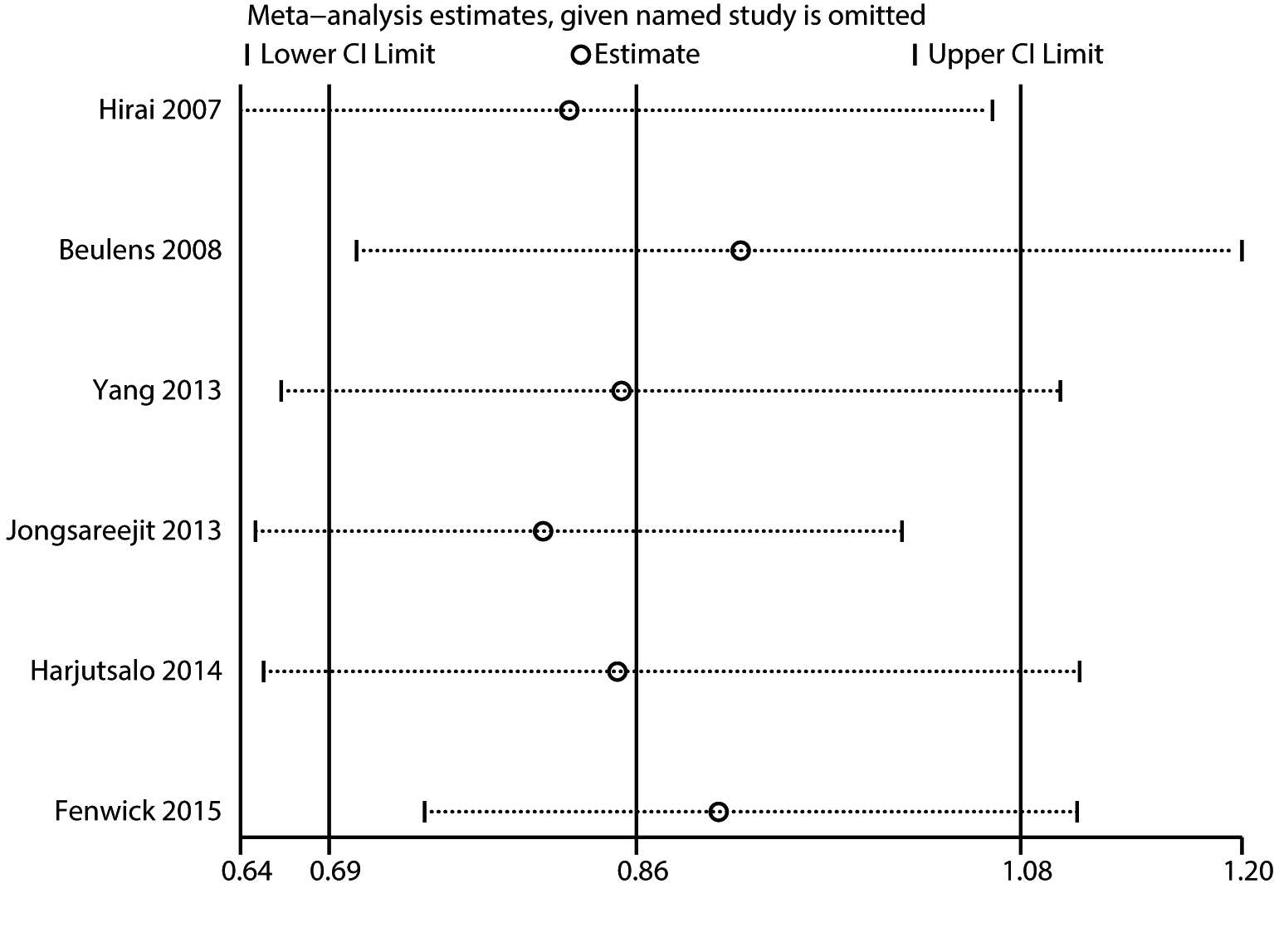

Supplement: Supplementary file 3 — Additional file 3. Sensitivity for cross-section studies. [file 12902_2020_588_MOESM3_ESM.tif]
